# 113 Impact of State Labor and Industry Benefits on Quality-Adjusted Life-Years Among Working Burn-Injured Adults

**DOI:** 10.1093/jbcr/irad045.086

**Published:** 2023-05-15

**Authors:** Caitlin Orton, Xinyao deGrauw, Barclay Stewart, Kushang Patel, Gretchen Carrougher, Karen Kowalske, Alyssa Bamer, April Hamilton, Jeffrey Schneider, Alice Ellyson

**Affiliations:** University of Washington, Seattle, Washington; University of Washington, Olympia, Washington; University of Washington, Seattle, Washington; University of Washington, Seattle, Washington; University of Washington, Seattle, Washington; UT Southwestern Medical Center, Dallas, Dallas, Texas; University of Washington, Golden, Colorado; University of Washington, Seattle, Washington; Spaulding Rehabilitation Hospital/Harvard Medical School/Rehabilitation Outcomes Center at Spaulding, Boston, Massachusetts; University of Washington, Seattle, Washington; University of Washington, Seattle, Washington; University of Washington, Olympia, Washington; University of Washington, Seattle, Washington; University of Washington, Seattle, Washington; University of Washington, Seattle, Washington; UT Southwestern Medical Center, Dallas, Dallas, Texas; University of Washington, Golden, Colorado; University of Washington, Seattle, Washington; Spaulding Rehabilitation Hospital/Harvard Medical School/Rehabilitation Outcomes Center at Spaulding, Boston, Massachusetts; University of Washington, Seattle, Washington; University of Washington, Seattle, Washington; University of Washington, Olympia, Washington; University of Washington, Seattle, Washington; University of Washington, Seattle, Washington; University of Washington, Seattle, Washington; UT Southwestern Medical Center, Dallas, Dallas, Texas; University of Washington, Golden, Colorado; University of Washington, Seattle, Washington; Spaulding Rehabilitation Hospital/Harvard Medical School/Rehabilitation Outcomes Center at Spaulding, Boston, Massachusetts; University of Washington, Seattle, Washington; University of Washington, Seattle, Washington; University of Washington, Olympia, Washington; University of Washington, Seattle, Washington; University of Washington, Seattle, Washington; University of Washington, Seattle, Washington; UT Southwestern Medical Center, Dallas, Dallas, Texas; University of Washington, Golden, Colorado; University of Washington, Seattle, Washington; Spaulding Rehabilitation Hospital/Harvard Medical School/Rehabilitation Outcomes Center at Spaulding, Boston, Massachusetts; University of Washington, Seattle, Washington; University of Washington, Seattle, Washington; University of Washington, Olympia, Washington; University of Washington, Seattle, Washington; University of Washington, Seattle, Washington; University of Washington, Seattle, Washington; UT Southwestern Medical Center, Dallas, Dallas, Texas; University of Washington, Golden, Colorado; University of Washington, Seattle, Washington; Spaulding Rehabilitation Hospital/Harvard Medical School/Rehabilitation Outcomes Center at Spaulding, Boston, Massachusetts; University of Washington, Seattle, Washington; University of Washington, Seattle, Washington; University of Washington, Olympia, Washington; University of Washington, Seattle, Washington; University of Washington, Seattle, Washington; University of Washington, Seattle, Washington; UT Southwestern Medical Center, Dallas, Dallas, Texas; University of Washington, Golden, Colorado; University of Washington, Seattle, Washington; Spaulding Rehabilitation Hospital/Harvard Medical School/Rehabilitation Outcomes Center at Spaulding, Boston, Massachusetts; University of Washington, Seattle, Washington; University of Washington, Seattle, Washington; University of Washington, Olympia, Washington; University of Washington, Seattle, Washington; University of Washington, Seattle, Washington; University of Washington, Seattle, Washington; UT Southwestern Medical Center, Dallas, Dallas, Texas; University of Washington, Golden, Colorado; University of Washington, Seattle, Washington; Spaulding Rehabilitation Hospital/Harvard Medical School/Rehabilitation Outcomes Center at Spaulding, Boston, Massachusetts; University of Washington, Seattle, Washington; University of Washington, Seattle, Washington; University of Washington, Olympia, Washington; University of Washington, Seattle, Washington; University of Washington, Seattle, Washington; University of Washington, Seattle, Washington; UT Southwestern Medical Center, Dallas, Dallas, Texas; University of Washington, Golden, Colorado; University of Washington, Seattle, Washington; Spaulding Rehabilitation Hospital/Harvard Medical School/Rehabilitation Outcomes Center at Spaulding, Boston, Massachusetts; University of Washington, Seattle, Washington; University of Washington, Seattle, Washington; University of Washington, Olympia, Washington; University of Washington, Seattle, Washington; University of Washington, Seattle, Washington; University of Washington, Seattle, Washington; UT Southwestern Medical Center, Dallas, Dallas, Texas; University of Washington, Golden, Colorado; University of Washington, Seattle, Washington; Spaulding Rehabilitation Hospital/Harvard Medical School/Rehabilitation Outcomes Center at Spaulding, Boston, Massachusetts; University of Washington, Seattle, Washington; University of Washington, Seattle, Washington; University of Washington, Olympia, Washington; University of Washington, Seattle, Washington; University of Washington, Seattle, Washington; University of Washington, Seattle, Washington; UT Southwestern Medical Center, Dallas, Dallas, Texas; University of Washington, Golden, Colorado; University of Washington, Seattle, Washington; Spaulding Rehabilitation Hospital/Harvard Medical School/Rehabilitation Outcomes Center at Spaulding, Boston, Massachusetts; University of Washington, Seattle, Washington

## Abstract

**Introduction:**

State labor and industry benefits (L&I) include workers’ compensation, wage replacement, vocational rehabilitation (VcR), workplace accommodations, job retraining, and labor protections. These vital services are not available to working adults injured outside their workplaces. We aimed to examine the impact of L&I on quality-adjusted life-years (QALY) lost among working adults with burn injury.

**Methods:**

Burn Model System National Database was queried for adult participants who self-identified as worker, volunteer, or caretaker and who answered the Veterans RAND 12-Item Health Survey (VR-12) at discharge (pre-injury recall) and 6-, 12- and 24-month post-injury. Participants were stratified into those with and without L&I and compared using independent samples t-test and Chi-squared test. Primary outcome, QALYs lost, was calculated by transforming VR-12 responses to SF-6D (SF-12) utility values. Mixed effects linear regression modeling was used to assess the impact of L&I on utility values over a 2-year period post-injury. Models were adjusted for age, sex, race, substance use, chronic medical conditions, burn size, hand injury, inhalation injury, and number of surgeries.

**Results:**

495 participants were analyzed (90 with L&I, 405 without L&I). Males accounted for 87% of L&I beneficiaries and 72% of those without L&I (p=0.001). No significant difference was seen in the other covariates. Mean utility values were 0.73 for L&I beneficiaries and 0.73 for those without L&I (p=0.99) pre-injury and were significantly lower for both groups at each timepoint post-injury (Figure 1). Greater utility value loss was seen in those with L&I compared to those without L&I at 6-month (p< =0.07), 12-month (p=0.02), and 24-month (p=0.03). During the 2 years post-injury, L&I beneficiaries lost 0.26 QALY and those without L&I lost 0.18 QALY (p=0.03).

**Conclusions:**

All working adults experienced a drop in utility values post-injury. Those who received L&I benefits lost more QALYs than their non-L&I peers. These findings are consistent with investigations among working adults with non-burn upper extremity and back injuries, suggesting there is opportunity to improve the delivery of L&I benefits and VcR for all injured workers.

**Applicability of Research to Practice:**

Current L&I benefits and VcR services for working adults with burn injury may not meet their needs. To actualize the benefits of VcR service delivery, the intensity of interventions should be more tailored to person-specific needs, risks of complicated return-to-work and recovery journeys.

